# The Sleep Symptoms Are Directly Associated With Suicide Risk in Adolescents and Youth Patients With Depression

**DOI:** 10.1155/da/2231053

**Published:** 2026-02-27

**Authors:** Yingying Zheng, Shuai Yuan, Jie Zhang, Yarong Ma, Hongbo He

**Affiliations:** ^1^ The Affiliated Brain Hospital of Guangzhou Medical University, Guangzhou, China; ^2^ School of Health Management, Guangzhou Medical University, Guangzhou, China, gzhmc.edu.cn; ^3^ Guangdong Engineering Technology Research Center for Translational Medicine of Mental Disorders, Guangzhou, China; ^4^ Shenzhen Second People’s Hospital, Shenzhen, China, szrch.com; ^5^ Guangdong Mental Health Center, Guangdong Provincial People’s Hospital, Guangzhou, China, gdghospital.org.cn

## Abstract

**Background:**

Multiple studies on adults have shown that depressive symptoms are the main contributing factors to suicide risk, and sleep symptoms are a major risk factor for depression. However, the associations between the three in adolescents have not been thoroughly studied. This study aims to examine the independent impact of sleep symptoms on suicide risk in adolescent depression and how this impact varies by age.

**Methods:**

Adolescent and youth (ages 13–25) depressed patients were included based on a depression cohort. Sleep symptoms were assessed using the Athens Insomnia Scale (AIS) and the Epworth Sleepiness Scale (ESS), depressive symptoms using the 17‐item Hamilton Depression Rating Scale (HAMD‐17), and suicide risk using the Beck Scale for Suicide Ideation (BSSI). A mediation model was employed to distinguish direct associations from pathways mediated by depression, with age as a moderator.

**Results:**

Among 744 depressed patients (mean age 18.31, 76.6% female), insomnia (*β* = 1.817, 95% CI: 1.505‐2.129) and hypersomnia (*β* = 1.344, 95% CI: 1.073‐1.615) had stronger direct associations on suicide risk than the depression‐mediated pathway. Younger patients (≤15 years) exhibited the strongest insomnia‐suicide associations (*β* = 1.950, 95% CI: 1.564‐2.335), compared to age 16–18 (*β* = 1.662, 95% CI: 1.363‐1.960) and age 19–25 (*β* = 1.278, 95% CI: 0.862‐1.693).

**Conclusions:**

Sleep symptoms (insomnia and hypersomnia) directly elevate suicide risk, exerting a stronger influence than depressive symptoms. This association is most pronounced in younger patients, particularly early adolescents (≤15 years), highlighting the critical need for early, sleep‐focused interventions in youth suicide prevention.

## 1. Introduction

Suicide is a critical global public health concern and ranks as the fourth leading cause of death among adolescents and youths (ages 15–29), accounting for over 700,000 deaths annually [1]. Among individuals who attempt suicide, ~80.8% have a diagnosable mental disorder, with major depressive disorder (MDD) representing the most significant risk factor [2]. In China, depressive disorders represent the second most prevalent cause of years lived with disability [3], with onset typically occurring around the age of 14 across most subtypes. In particular, an estimated 280,000 people in China die by suicide each year, with depression accounting for around 40% of these deaths [4]. Previous research has identified a wide range of social, psychological, and physiological factors associated with suicide risk [5–8]. A meta‐analysis demonstrated that mood disorders, including depression, are strongly associated with suicide [9]. Furthermore, longitudinal studies suggest that nearly one in six individuals diagnosed with MDD eventually die by suicide [10]. Despite advancements in the treatment of depression, the World Health Organization (WHO) reports that global suicide rates have not shown a substantial decrease, and rates are set to rise over 15 years [1].

Previous studies have indicated that sleep symptoms are highly prevalent among individuals with MDD [11, 12]. It is uncommon for patients experiencing a major depressive episode to avoid some form of sleep‐related symptomatology. Insomnia, in particular, is the most frequently reported sleep complaint among individuals with comorbid depression, affecting ~80%–90% of patients [13]. Sleep quality is linked to mental health. Research shows that sleep disturbances can lead to suicidal thoughts and behavior [14, 15]. There is consistent research indicating that sleep disturbances, including both short and long sleep duration, as well as insomnia, are associated with an elevated suicide risk [16], manifesting in a higher likelihood of reporting suicide ideation (SI) or a history of suicide attempts (SA) [17]. Large‐scale epidemiological studies have specifically identified short sleep duration as a significant correlate of both SI and SA [18]. Furthermore, the intricacy of this relationship has been emphasized by researchers, who have reported a nonlinear association between sleep duration and suicide risk. This means the longer someone sleeps, the higher the risk of suicide and self‐harm [19]. However, meta‐analytic findings suggest that hypersomnia may not have a significant association with subsequent SI, introducing inconsistencies into the literature [20]. Taken together, while sleep disturbances appear to be a critical factor in suicide risk, the nature of these associations remains unclear. Notably, the majority of existing studies have focused on adult populations, with relatively limited research targeting adolescents and young adults—a critical developmental period when early intervention could have lasting impacts. Future studies are urgently needed to clarify these associations and to explore developmental nuances in younger populations.

Ages 13–25 represent a critical developmental period marked by simultaneous physical and psychological maturation [21, 22]. During this stage, adolescents and young adults increasingly report poor sleep quality, characterized by difficulties initiating sleep, maintaining sleep, and experiencing frequent nocturnal awakenings [9, 23, 24]. These sleep disruptions often lead to impaired daytime functioning, including excessive sleepiness and cognitive deficits. A recent survey found that the average amount of sleep teenagers get is 7.25 h, with half of them getting insufficient sleep. Importantly, a clear nonlinear association between sleep symptoms and suicide risk in teenagers has been observed, with depression identified as a potential mediator [15]. Longitudinal studies further suggest that sleep problems during adolescence may increase the risk of suicide by heightening negative emotions, impairing self‐regulation, and promoting nonsuicidal self‐injury [25]. Irregular sleep patterns and circadian rhythm disturbances have also been shown to adversely impact behavior, mood regulation, and attentional control [26]. A study conducted in Korea indicated that circadian misalignment, stemming from discrepancies between weekday and weekend sleep schedules, may be linked to poor mental health outcomes among adolescents [27]. Additionally, previous research by our team has shown that sleep symptoms, particularly daytime dysfunction, are significantly correlated with increased suicidal ideation in clinically depressed adolescents [28]. While an increasing body of research has explored the association between sleep disturbances and suicide risk, much of the existing literature has focused on the general adolescent population, with relatively limited attention given to clinically depressed adolescents. Moreover, there remains a notable gap in understanding the relationship between hypersomnia and suicide risk.

Clarifying whether, and which, sleep symptoms are associated with elevated suicide risk in depressed adolescents—and how these associations evolve with age—may enhance clinicians’ attention to the critical need for early, sleep‐focused interventions in adolescent and youth suicide prevention. The present study aims to investigate the dual independent pathways through which sleep symptoms are linked to suicide risk. We hypothesize that sleep symptoms (insomnia and hypersomnia) are not only associated with more severe depressive symptoms—which may be indirectly linked to elevated suicide risk—but also show an independent, direct association with suicide risk. Furthermore, we propose that the direct association between sleep symptoms and suicide risk follows a nonlinear pattern, characterized by a gradual weakening with increasing age.

## 2. Methods

### 2.1. Study Design and Participants

This multicenter, cross‐sectional observational study enrolled patients from five different hospitals in Guangdong Province, South China, including Shenzhen Kangning Hospital, Foshan Shunde Wu Zhongpei Memorial Hospital, Guangdong Provincial Hospital of Traditional Chinese Medicine, Jiangmen Third People’s Hospital, and the Affiliated Brain Hospital of Guangzhou Medical University. Participants were interviewed within 3 days after admission or during the first visit to the outpatient clinic between January 2022 and September 2023 and were considered for inclusion. All patients were clinically diagnosed with depression according to the International Classification of Diseases, Tenth Revision (ICD‐10) by their admitting physician and underwent a comprehensive assessment, including demographic and medical history, social factors, psychological health, and clinical presentation. A total of 744 young depressed patients aged 13–25 years were finally included. Written informed consent was obtained from the participants or their guardians for those under 18 years of age. The protocol was approved by the Ethics Committee of the Affiliated Brain Hospital of Guangzhou Medical University.

### 2.2. Measures

Patients were assessed for suicidality, depression, daytime sleepiness, and nighttime insomnia. Using a self‐administered questionnaire, demographic information was collected: sex, age, marital status, level of education, anthropometric measures (height and weight), substance use (smoking and alcohol consumption), family history of mental illness, and comorbid physical conditions.

The patient’s depressive symptoms were assessed using the 17‐item Hamilton Depression Rating Scale (HAMD‐17) developed by Hamilton et al. in 1960. The assessment encompasses five domains: anxiety/somatization, weight loss, cognitive impairment, psychomotor retardation, and sleep disturbances. Each item is evaluated on a scale of 0–4 or 0–2, with higher scores indicating more severe depressive symptoms [29]. Sleep symptoms were assessed primarily for insomnia and hypersomnia, with the Athens Insomnia Scale (AIS) for insomnia and the Epworth Sleepiness Scale (ESS) for hypersomnia. The AIS was developed by Thomas Roth et al. in 1985. It is a scale that measures the severity of insomnia on a 4‐point scale (0–3), ranging from none to severe. Higher scores indicate more severe insomnia [30]. The ESS is a 4‐point scale (0–3) ranging from no drowsiness to a high probability of drowsiness. It is important to note that higher cumulative scores are indicative of a greater severity of daytime sleepiness [31]. The patient’s suicide risk was assessed using the Beck Scale for Suicide Ideation (BSSI). This scale was developed by Aaron T. Beck and colleagues in 1979. It is used to assess the frequency, duration, and intensity of suicidal thoughts. Scores of 9 or above indicate high‐risk thresholds requiring clinical intervention [32].

### 2.3. Statistical Analyses

Descriptive statistics were employed to summarize the sociodemographic and clinical characteristics of the participants. For continuous variables (age, body mass index (BMI), insomnia, hypersomnia, depressive symptoms, suicide risk, and anxiety symptoms), means (M) and standard deviations (SD) were calculated. For categorical variables (sex, years of education, aggression, family history of mental illness, presence of physical ailments, smoking status, and alcohol use), frequencies (n) and percentages (%) were reported. Bivariate relationships were examined using Pearson’s correlation coefficients.

**Figure 1:**
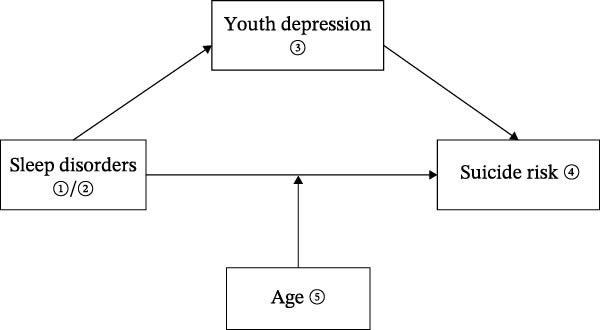
Graphical representation of a mediation model from sleep symptoms to suicide risk.

Two independent mediation models were constructed—one for insomnia and one for hypersomnia. In each model, sleep symptoms were specified as the predictor variable, suicide risk as the outcome variable, and depressive symptoms as the mediating variable. Using 5,000 resample bias‐corrected bootstrap mediation analyses, the total effect was partitioned into direct and indirect effects, and 95% confidence intervals (CI) were calculated to determine statistical significance. Building on these mediation models (Figure 1), a moderated mediation model was subsequently developed to examine whether age moderates the direct association between sleep symptoms and suicide risk. Conditional direct effects of sleep symptoms on suicide risk were estimated at three developmental stages: early adolescence (≤15 years; 25th percentile), the mean age (18 years), and young adulthood (22 years; 75th percentile).

Models included sex, age, anxiety symptoms, family history of mental illness, and physical comorbidities as control variables to account for potential confounding. Statistical analyses were performed using IBM SPSS Statistics (version 26.0; SPSS Inc., Chicago, USA) with the PROCESS macro (version 4.0) to test the hypothesized pathways.

## 3. Results

### 3.1. Sample Characteristics

The clinical cohort consisted of 744 depressed adolescents and youths, with a mean age of 18.31 ± 3.28 years, and 76.61% were female. The cohort presented moderate symptom severity across various domains: insomnia (AIS = 11.12 ± 5.50), hypersomnia (ESS = 9.72 ± 5.63), depression (HAMD‐17 = 18.16 ± 8.10), and suicide risk (BSSI = 36.49 ± 23.72). Educational levels were diverse, with 36.96% having a college‐level education and 40.32% having completed senior high school. Physical comorbidities were present in 14.92% of the sample, while 19.49% had a family history of psychiatric disorders (Table 1).

**Table 1:** Sociodemographic profile of depressed patients (*N* = 744).

Characteristic	Categorization	Total participants
Age, y, mean (SD)	NA	18.31 (3.28)

Sex, *n* (%)	Male	174 (23.39)
	Female	570 (76.61)

Years of education, *n* (%)	Primary school or lower	6 (0.81)
	Junior high school	163 (21.91)
	Senior high school	300 (40.32)
	College or higher	275 (36.96)

Marital status, *n* (%)	In a relationship	153 (20.56)

^∗^BMI, y, mean (SD)	NA	20.95 (4.38)

With physical ailments, *n* (%)	Yes	111 (14.92)

Aggression, *n* (%)	Yes	20 (2.69)

Family history of mental illness, *n* (%)	Yes	145 (19.49)

Smoking status, *n* (%)	Yes	67 (9.01)

Alcohol status, *n* (%)	Yes	55 (7.39)

^a^AIS, mean (SD)	NA	11.12 (5.50)

^b^ESS, mean (SD)	NA	9.72 (5.63)

^c^HAMD‐17, mean (SD)	NA	18.16 (8.10)

^d^HAMA‐14, mean (SD)	NA	19.93 (9.49)

Suicide risk, mean (SD)	NA	36.49 (23.72)

^a^AIS, the assens insomnia scale.

^b^ESS, the epworth sleepiness scale.

^c^HAMD‐17, the 17‐hamilton depression scale.

^d^HAMA‐14, the 14‐hamilton anxiety scale

^∗^Body mass index.

### 3.2. Correlation Analysis

Bivariate analyses revealed significant positive correlations between sleep symptoms, depressive symptoms, suicide risk, and age across the cohort (all *p* < 0.001). Insomnia demonstrated the strongest correlation with suicidality (*r* = 0.526), followed by depressive symptoms (*r* = 0.422), and hypersomnia (*r* = 0.415). It is noteworthy that age was significantly negatively correlated with insomnia (*r* = −0.169), hypersomnia (*r* = −0.222), depressive symptoms (*r* = −0.105), and suicide risk (*r* = −0.349) (Table 2).

**Table 2:** Correlation matrix of sleep symptoms, depression, suicide risk, and age (*N* = 744).

Variables	1	2	3	4	5
1. Insomnia^a^	1	0.441 ^∗∗∗^	0.553 ^∗∗∗^	0.526 ^∗∗∗^	−0.169 ^∗∗∗^
2. Hypersomnia^b^	—	1	0.292 ^∗∗∗^	0.415 ^∗∗∗^	−0.222 ^∗∗∗^
3. Depression^c^	—	—	1	0.422 ^∗∗∗^	−0.105 ^∗∗∗^
4. Suicide Risk^d^	—	—	—	1	−0.349 ^∗∗∗^
5. Age	—	—	—	—	1

^a^AIS, the assens insomnia scale.

^b^ESS, the epworth sleepiness scale.

^c^HAMD‐17, the 17‐Hamilton depression scale.

^d^BSSI, beck scale for suicide ideation.

^∗^ *p* < 0.05.

^∗∗^ *p* < 0.01.

^∗∗∗^ *p* < 0.001.

### 3.3. Mediation Analysis

The mediation models revealed distinct pathways through which sleep symptoms influenced suicide risk. For insomnia (Figure 2a1), the total effect on suicide risk was significant (*β* = 2.267, 95% CI [2.003, 2.532], *p* < 0.001), with the majority of this effect operating independently of depression. Direct pathways accounted for 80.15% of insomnia’s total impact (*β* = 1.817, 95% CI [1.505, 2.129], *p* < 0.001), while depression mediated only 19.85% of the association (*β* = 0.450, 95% CI [0.271, 0.647], *p* < 0.001). In Model 2, where sex, age, anxiety symptoms, family history of mental illness, and physical comorbidities were controlled for, the direct effect of sleep symptoms on suicide risk increased to 94.48% (*β* = 1.633, 95% CI [1.332, 1.935], *p*  < 0.001) (Figure 2a2).

Figure 2The graphical model of mediation for sleep symptoms, depression, and suicide risk. (a1, b1), uncontrolled confounding factors; (a2, b2), included sex, age, anxiety symptoms, family history of mental illness, and physical comorbidities as control variables. ① AIS, the assens insomnia scale; ② ESS, the epworth sleepiness scale; ③ HAMD‐17, the 17‐hamilton depression scale; ④ Suicide risk.

**Figure:**
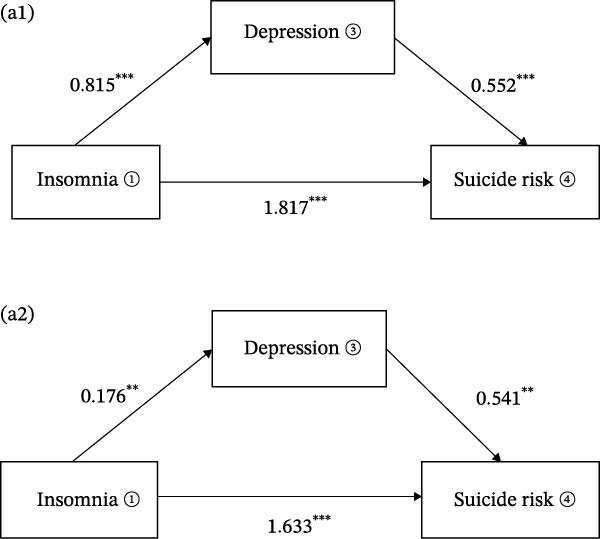
(a)

**Figure:**
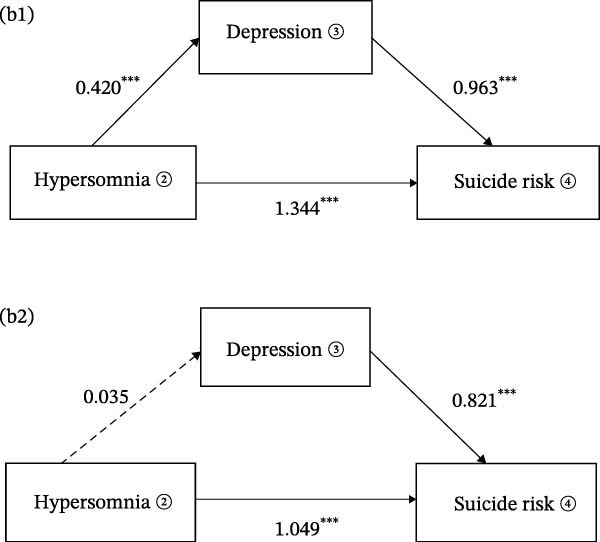
(b)

Similarly, hypersomnia(Figure 2b1) exhibited a robust total effect on suicide risk (*β* = 1.748, 95% CI [1.472, 2.024], *p* < 0.001), with direct mechanisms explaining 76.89% of its influence (*β* = 1.344, 95% CI [1.073, 1.615], *p* < 0.001) and depression mediating 23.11% (*β* = 0.404, 95% CI [0.285, 0.540], *p* < 0.001). However, after controlling for confounding variables(Figure 2b2), the mediation effect was not significant (*β* = 10.029, 95% CI [−0.016,0.079], *p* = 0.240), and the direct effect of hypersomnia on suicide risk was strengthened (*β* = 1.049, 95% CI [0.781, 1.317], *p* < 0.001) (Table 3).

**Table 3:** Mediation analysis of sleep symptoms on suicide risk: the role of depression (*N* = 744).

Effect	Path	*β* (SE)	Effect size as % of the total effect	95% CI		*p*
				LLCI	ULCI	
Model 1						
Total effect	Insomnia	2.267	100.00	2.003	2.532	<0.001
	Hypersomnia	1.748	100.00	1.472	2.024	<0.001
Direct effect	①→④	1.817	80.15	1.505	2.129	<0.001
	②→④	1.344	76.89	1.073	1.615	<0.001
Indirect effect	①→③→④	0.450	19.85	0.271	0.647	<0.001
	②→③→④	0.404	23.11	0.285	0.540	<0.001
Model 2^a^						
Total effect	Insomnia	1.729	100.00	1.431	2.027	<0.001
	Hypersomnia	1.078	100.00	0.806	1.350	<0.001
Direct effect	①→④	1.633	94.48	1.332	1.935	<0.001
	②→④	1.049	97.31	0.781	1.317	<0.001
Indirect effect	①→③→④	0.096	5.52	0.035	0.036	<0.001
	②→③→④	0.029	2.69	−0.016	0.079	0.240

*Note:* ① AIS, the assens insomnia scale; ② ESS, the epworth sleepiness scale; ③ HAMD‐17, the 17‐hamilton depression scale; ④ Suicide risk.

^a^Models included sex, age, anxiety symptoms, family history of mental illness, and physical comorbidities as control variables.

Age moderated these pathways in a sleep‐specific manner. Younger patients (early adolescents, ≤15 years) exhibited the strongest insomnia‐suicide associations (*β* = 1.950, 95% CI [1.564, 2.335]), compared to those aged 16–18 (*β* = 1.662, 95% CI [1.363, 1.960]) and 19–25 (*β* = 1.278, 95% CI [0.862, 1.693]). The indirect effect of depression was weak but significant (*β* = 0.270, 95% CI [0.270, 0.621]). In the hypersomnia model, the correlation between hypersomnia and suicide risk also showed a decreasing trend with age, but the mediating role of depression was not significant (*β* = 0.308, 95% CI [−0.016, 0.081]). Even after controlling for confounding variables, both models displayed similar results (Table 4; Supporting Information).

**Table 4:** Differential age effects in the mediation pathways from sleep symptoms to suicidal risk (*n* = 744).

Effect	Path	*β* (SE)	95% CI		*p*
			LLCI	ULCI	
Insomnia model					
Conditional direct effect	①→④	—	—	—	—
−15 years old ^∗^	—	1.950 (0.196)	1.564	2.335	＜0.001
−18 years old ^∗^	—	1.662 (0.153)	1.363	1.960	＜0.001
−22 years old ^∗^	—	1.278 (0.212)	0.862	1.693	＜0.001
Indirect effect	①→③→④	0.440 (0.091)	0.270	0.621	0.002
Hypersomnia model					
Conditional direct effect	②→④	—	—	—	—
−15 years old ^∗^	—	1.269 (0.179)	0.918	1.619	＜0.001
−18 years old ^∗^	—	1.110 (0.135)	0.844	1.375	＜0.001
−22 years old ^∗^	—	0.898 (0.216)	0.473	1.323	＜0.001
Indirect effect	②→③→④	0.387 (0.061)	0.274	0.511	0.092

*Note:* ① AIS, the assens insomnia scale; ② ESS, the epworth sleepiness scale; ③ HAMD‐17, the 17‐hamilton depression scale; ④ Suicide risk; ⑤ Age.

^∗^Age values represent distribution landmarks: 15 years (25th percentile), 18 years (sample mean), and 22 years (75th percentile).

## 4. Discussion

This study provides a preliminary exploration of the association between sleep symptoms (insomnia and hypersomnia) and suicide risk. Our findings suggest that sleep symptoms play a crucial role in the onset and progression of suicide risk among adolescents and young adults. Insomnia and hypersomnia not only exacerbate depressive symptoms, thereby increasing suicide risk, but also directly contribute to a heightened risk of suicide. Additionally, our results indicate that the impact of insomnia on suicide risk is more pronounced in younger adolescents (≤15 years).

Previous studies have suggested that insomnia directly influences elevated suicidal ideation and behavior, which aligns with our findings, while the impact of daytime sleepiness has been more variable. In our study, insomnia had a more significant direct association with suicide risk than hypersomnia. From a neuroimaging perspective, this may be due to insomnia’s strong association with impaired function of the left dorsolateral prefrontal cortex (LDLPFC) [33], which plays a crucial role in cognitive functions, particularly response inhibition and conflict control [34]. Chronic insomnia disorder (CID) reduces the prefrontal cortex’s (PFC) ability to regulate the amygdala, leading to its hyperactivation [35, 36]. This hyperactivation is not only linked to depressive symptoms but may also contribute to increased impulsive behavior [37]. In adolescents and young adults, whose PFC is still undergoing remodeling [38], the associations of insomnia with impulsive behavior are likely to be more pronounced. Additionally, nighttime wakefulness tends to exacerbate the consolidation of negative memories, creating a “Mind After Midnight” effect. This, combined with heightened impulsivity, may explain why many SA occur during the early morning hours [39].

The direct associations of insomnia on suicide risk were found to be stronger than the mediating effect of depression, and these associations were particularly pronounced in younger patients, a finding that has been less frequently discussed in previous studies. Adolescence is a critical period for the development of both the PFC and the limbic system, which are essential for emotion regulation and impulse control [22]. Sleep deprivation has been shown to impair PFC function, leading to increased amygdala activation and a heightened vulnerability to negative emotions [40, 41]. Adolescents who slept less than 6 h per night (compared to 8 h) had more than three times the odds of contemplating suicide, planning an attempt, or actually attempting suicide. Furthermore, they had over four times the odds of experiencing an attempt that required medical intervention [42, 43]. As the adolescent brain is still undergoing development, imbalances in neural circuits related to emotion regulation and impulse control increase the likelihood of impulsive suicidal behavior. Sleep deprivation also reduces the protein brain‐derived neurotrophic factor (BDNF) levels, further impairing emotion regulation [44]. Adolescents are exposed to a range of stressors, such as academic pressures and social challenges, and sleep deprivation diminishes their capacity to cope with these stressors [45]. When sleep efficiency declines, working memory also suffers, leading to reduced academic performance and increased frustration, thus creating a “stress‐insomnia‐dysfunction” cycle [46]. This cycle exacerbates feelings of hopelessness and further elevates the risk of suicide among sleep‐deprived adolescents. Given their neurodevelopmental stage and the unique social pressures they face, adolescents are particularly vulnerable to sleep problems that can lead to suicidal behavior. Therefore, suicide prevention efforts for adolescents and youths should focus on improving sleep hygiene and incorporating cognitive–behavioral interventions to disrupt this harmful cycle.

Despite the significant contributions of this study, several limitations should be noted. First, the sample was restricted to clinically depressed adolescents, without comparison to other demographic or socioeconomic groups, which may limit the generalizability of the findings. Second, the cross‐sectional design precludes the ability to assess temporal changes in the relationship between sleep disorders and suicide risk and prevents causal inferences. Third, insufficient control of confounding variables, such as medication use, socioeconomic status, and life stressors, may have exaggerated the observed associations. Fourth, the use of the ESS to assess hypersomnia has limitations, as it measures subjective daytime sleepiness rather than clinically defined hypersomnia (i.e., prolonged sleep duration), and its validity in depressed adolescents remains unestablished, potentially limiting the accuracy of assessment. Future research should focus on longitudinal tracking of sleep patterns in depressed adolescents, integrate additional risk factors, and refine suicide risk models. The use of multidimensional tools, such as the sleep duration dimension of the Pittsburgh Sleep Quality Index, polysomnography, or specialized hypersomnia scales, could improve the precision of excessive sleep symptom measurement and further validate the link between hypersomnia and suicide risk.

## 5. Conclusion

The present study reveals that sleep symptoms—including insomnia and hypersomnia—have a direct and independent association with suicidal risk, exceeding the predictive power of depressive symptoms in a clinical adolescent and youth group. Age‐related associations of sleep symptoms: Sleep symptoms in early adolescents (≤15 years of age) have an even greater impact on suicide risk compared to youths. Given this, there is a need to pay greater attention to the role of sleep issues in suicide prevention strategies, especially for the adolescent population.

## Author Contributions

All authors have significantly contributed to this work. Each author actively participated in conceiving and refining the manuscript’s objectives, conducting literature reviews, and crafting and refining the manuscript. **Yingying Zheng and Shuai Yuan**: writing – original draft preparation, formal analysis. **Jie Zhang and Yarong Ma**: writing – review, data curation. **Hongbo He**: methodology, supervision.

## Funding

This study was supported by the Guangzhou Science and Technology Bureau City‐University Union Funding (Grants 2023A03J0844, 2023A03J0434, 2023A03J0845), Guangzhou Key Laboratory of Psychosomatic Medicine (Grant SL2023A03J00421), and Guangzhou Key Clinical Specialty (Clinical Medical Research Institute).

## Ethics Statement

This study was conducted in accordance with the principles of the Declaration of Helsinki. Written informed consent was obtained from all participants and their legal guardians. The study protocol was reviewed and approved by the Ethics Committee of the Affiliated Brain Hospital of Guangzhou Medical University (AF/SC‐08/02.3).

## Conflicts of Interest

The authors declare no conflicts of interest.

## Supporting Information

Additional supporting information can be found online in the Supporting Information section.

## Supporting information


**Supporting Information** Differential age effects in the mediation pathways from sleep symptoms to suicidal risk (*n* = 744). The models included the following control variables: sex, age, anxiety symptoms, family history of mental illness, and physical comorbidities.

## Data Availability

Data will be made available from the corresponding author upon reasonable request.
