# Supplementary material for: The Sleep Symptoms Are Directly Associated With Suicide Risk in Adolescents and Youth Patients With Depression
Source: Depress Anxiety. 2026 Feb 27;2026:2231053. doi: 10.1155/da/2231053 (PMC12947251; doi:10.1155/da/2231053)
Supplement: Supplementary file 1 — Supporting Information Differential age effects in the mediation pathways from sleep symptoms to suicidal risk (n = 744). The models included the following control variables: sex, age, anxiety symptoms, family history of mental illness, and physical comorbidities. [file DA-2026-2231053-s001.docx]

**Supplementary table. Differential age effects in the mediation pathways from sleep symptoms to suicidal risk(*n*=744)**

| **Effect** | **Path** | ***β (SE)*** | **95% CI** | | ***p*** |
| --- | --- | --- | --- | --- | --- |
|  |  |  | **LLCI** | **ULCI** |  |
| **Insomnia Model** | | | | | |
| **Conditional Direct Effect** | ①→④ |  |  |  |  |
| -15 years old^*^ |  | 1.904 (0.198) | 1.564 | 2.335 | **＜0.001** |
| -18 years old^*^ |  | 1.653 (0.154) | 1.363 | 1.960 | **＜0.001** |
| -22 years old^*^ |  | 1.318 (0.212) | 0.862 | 1.693 | **＜0.001** |
| **Indirect Effect** | ①→③→④ | 0.099 (0.035) | 0.270 | 0.621 | **0.002** |
| **Hypersomnia Model** | | | | | |
| **Conditional Direct Effect** | ②→④ |  |  |  |  |
| -15 years old^*^ |  | 1.185 (0.181) | 0.918 | 1.619 | **＜0.001** |
| -18 years old^*^ |  | 1.045 (0.137) | 0.844 | 1.375 | **＜0.001** |
| -22 years old^*^ |  | 0.857 (0.216) | 0.473 | 1.323 | **＜0.001** |
| **Indirect Effect** | ②→③→④ | 0.308 (0.024) | -0.016 | 0.081 | 0.208 |

Models included sex, age, anxiety symptoms, family history of mental illness, and physical comorbidities as control variables.

*Age values represent distribution landmarks: 15 years (25th percentile), 18 years (sample mean), and 22 years (75th percentile).

① AIS, The Assens Insomnia Scale; ② ESS, The Epworth Sleepiness Scale; ③ HAMD-17,the 17-Hamilton Depression Scale ; ④Suicide Risk; ⑤ Age.
